# Association between occupational exposures to pesticides with heterogeneous chemical structures and farmer health in China

**DOI:** 10.1038/srep25190

**Published:** 2016-04-27

**Authors:** Xusheng Huang, Chao Zhang, Ruifa Hu, Yifan Li, Yanhong Yin, Zhaohui Chen, Jinyang Cai, Fang Cui

**Affiliations:** 1 Department of Neurology, Chinese PLA General Hospital, 28 Fuxing Road, Beijing 100853, P. R. China; 2 School of Management and Economics, Beijing Institute of Technology, 5 South Zhongguancun Street, Beijing 100081, P. R. China; 3 Department of Neurology, the First Affiliated Hospital of PLA General Hospital, No. 51 Fucheng Road, Beijing 100048, P. R. China

## Abstract

This study analyzed the associations of farmers’ exposure to organophosphates (OPs), organosulfurs (OSs), organonitrogens (ONs) and pyrethroids (PYRs) with parameters of the blood complete counts (CBC), a blood chemistry panel (BCP) and the conventional nerve conduction studies among 224 farmers in China in 2012. Two health examinations and a series of follow-up field surveys were conducted. Multiple linear regression analyses were used to evaluate the associations. The results show considerable associations between multiple groups of pesticides and several CBC parameters, but it was not enough to provide evidence of hematological disorders. The short- and medium-term OPs exposures were mainly associated with liver damage and peripheral nerve impairment, respectively, while OSs exposure might induce liver damage and renal dysfunction. The neurotoxicity of ONs was second only to OPs in addition to its potential liver damage and the induced alterations in glucose. In comparison, the estimated results show that PYRs would be the least toxic in terms of the low-dose application. In conclusion, occupational exposures to pesticides with heterogeneous chemical structures are associated with farmer health in different patterns, and the association between a specific group of pesticides and farmer health also differs between the short- and medium-term exposures.

Although pesticides are important for agricultural production throughout the world^1^, the widespread application of pesticides has induced well-documented adverse health effects in humans^2^,^3^,^4^,^5^,^6^,^7^. Most epidemiological studies have analyzed the health effects of exposure to a single pesticide or a specific group of pesticides with similar chemical structure, measuring exposure level by using the sum of all pesticides, or classifying the study subjects into exposed (or high-exposed) and non-exposed (or low-exposed) groups^8^,^9^,^10^,^11^,^12^,^13^. In addition, the previous studies have often analyzed the associations of pesticide exposure with human health using a single parameter or a group of parameters for a specific health type^14^,^15^,^16^,^17^.

In reality, the examined health parameters may be probably affected by a complex mixture of pesticides. For example, farmers in China not only use a wide variety but also a great amount of pesticides to protect their crops, which greatly differs from farmers in other regions^18^,^19^. However, few previous studies have associated human health with exposures to pesticides with heterogeneous chemical structures in an integrated framework, which would generate the biased results. Moreover, most previous studies have analyzed the associations of pesticide exposure with the risk ratio (RR), odds ratio (OR) and rate difference (RD). However, it should be noted that some irreversible sub-clinical damages to human health induced by pesticide exposure would also be unobservable. Unfortunately, few studies have focused on this important issue. This study aimed to analyze and compare the sub-clinical health effects of occupational exposures to pesticides with heterogeneous chemical structures among 224 farmers in China in 2012. Farmer health was measured using 52 parameters in terms of the complete blood cell counts (CBC), a blood chemistry panel (BCP), and the conventional nerve conduction studies (CNCS).

## Methods

### Study sample

Data for this study were collected from farmers in three Chinese provinces (Guangdong, Jiangxi and Hebei) in 2012, which has been previously described^11^. In brief, we randomly selected two counties within each province, two villages within each county, and 20–25 farmers within each village. An eligible farmer was defined as the person who was over 18 years old and mainly sprayed pesticides in the household. A total of 246 farmers were initially enrolled, but 22 farmers were excluded due to their failure to provide information of pesticide application or their absence from the health examinations. This study was approved by the Ethics Committee of Chinese PLA General Hospital, and all farmers provided informed consent before the health examinations. The methods in this study were carried out in accordance with the approved guidelines. A baseline face-to-face survey was conducted to collect data regarding the individual characteristics, which included household population, number of labors, history of farm activity and pesticide application, cropping structure, age, sex, smoking status, and alcohol consumption.

### Health examinations and outcomes

Two health examinations were performed by professional doctors and technicians from Chinese PLA General Hospital in 2012. The first examination was performed before farmers began planting crops in March, and the second examination was performed before the end of the crop harvest. A total of 52 health parameters regarding CBC, BCP and CNCS were examined (see Table S1). Moreover, farmers’ height and weight were also obtained at two health examinations.

A blood sample was collected in the morning after a 12-hour fast for each farmer. All blood samples were subsequently centrifuged and refrigerated until they were submitted for laboratory testing in Beijing within 8 hours. Moreover, all blood samples from the first and second examinations were tested at the same laboratory to ensure consistency.

The CNCS were performed using the surface electrodes with standard placements on the non-dominant side of the farmers. The upper and lower limbs were warmed to maintain temperatures of 32‒34 °C and 30‒33 °C, respectively. The CNCS parameters consisted of the motor conduction velocities, the distal motor latencies, and the compound muscle action potential amplitudes of the median, ulnar, tibial, and common peroneal nerves, as well as the sensory conduction velocities and the sensory action potential amplitudes of the median, ulnar, and sural nerves.

### Assessing pesticide exposure

Occupational pesticide exposures were measured using the amount (in kilograms) of the pesticides’ active ingredients applied by farmers. All farmers were asked to record information of all pesticides they applied. The essential information included the chemical name, the active ingredient percentage(s), the applied amount, and the purchase price of each applied pesticide, as well as the target crop, date, and duration of each pesticide application. To ensure the data accuracy and reliability, we provided several training sessions regarding how to make effective records, and conducted a follow-up visit semimonthly to check if they made accurate records. Note that all farmers were asked to keep the package bags or bottles of pesticide products for each follow-up check to minimize the recall biases. Using these data, we were able to calculate the amount of each active ingredient.

In this study, all pesticides were classified into organophosphates (OPs), organosulfurs (OSs), organonitrogens (ONs), pyrethroids (PYRs) and others according to the chemical structures of the active ingredients (see Table S2). The adopted standard criteria for classification were based on the recommendations from the Institute for the Control of Agrochemicals, Ministry of Agriculture of China^20^,^21^. To specify the associations of pesticide exposure with health parameters during different durations, pesticides applied within and more than 10 days before the second health examination was defined as the short- and medium-term exposure, respectively.

### Statistical analysis

In this study, multiple linear regression analyses were used to evaluate the associations between pesticide exposures and the health parameters. Both the short- and medium-term exposures for each group of pesticides were integrated in the multivariate model separately. The multivariate model was also adjusted for age, sex, body mass index, smoking status, alcohol consumption, and two province dummy variables. We included the baseline parameter from the first examination in the final multivariate model to control for systematic heterogeneity. Thus, the final model was described as:


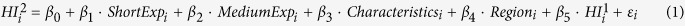


In equation (1), *HI*_*i*_^2^ and *HI*_*i*_^1^ indicate the health parameter of the second and first health examination of the *i*-th farmer, respectively. *ShortExp*_*i*_ *and MediumExp*_*i*_indicate farmers’ exposure in the short and medium term, respectively. *Characteristics*_*i*_ consists of age, sex, body mass index, smoking status and alcohol consumption. *Region*_*i*_ is a vector of dummies for provinces, including Guangdong and Jiangxi. *ε*_*i*_ indicates the random error term. *β*_0_–*β*_5_ indicate the coefficients to be estimated. Thus, equation (1) is similar to the first-order auto-regression model described in the econometric literature^22^.

All tests were two-tailed, and *p* < 0.05 was considered statistically significant. All statistical analyses were performed using STATA version 13.1 (StataCorp LP, College Station, Texas).

## Results

### Demographic characteristics and exposure levels

Table 1 summarizes the demographic characteristics, and levels of pesticide exposures. The final sample consisted of 224 farmers with a mean age of 51.77 years and a mean body mass index of 23.53 kg/m^2^. Among these farmers, 26.8% were female, 46.9% had a smoking habit, and 42.4% consumed alcohol.

As described in Table 1, the sample farmers applied an average of approximately 4.54 kg of pesticides between two health examinations. In the short term, the average amount of OPs and OSs was 0.09 kg and 0.08 kg, respectively, higher than the amounts of ONs, PYRs and other pesticides. In the medium term, the average amount of OPs was approximately 2.11 kg, which was the largest amount among these five groups of pesticides. The average amount of ONs was 1.15 kg, which was second to OPs but larger than that of OSs (0.51 kg) and other pesticides (0.41 kg). Among ONs, the most commonly used pesticides were carbamate and neonicotinoid insecticides. In addition, PYRs remained the least applied group of pesticides with an average amount of 0.07 kg.

### Associations of pesticide exposures with the complete blood cell counts

Table 2 reports the associations between pesticide exposures and CBC parameters. None of the CBC parameters was associated with OPs exposure in either short or medium terms. The decreased platelet distribution width (PDW) was associated with the short-term OSs and PYRs exposures. Following the medium-term exposure, the increased PDW was positively associated with PYRs, but insignificantly associated with OSs. In terms of the white blood cells, each kilogram increase in the medium-term OSs exposure was associated with an increase in lymphocyte counts (Lym) of 0.07 × 10^9^/L (95% CI: 0.00, 0.14). The short-term ONs exposure was also associated with the increased Lym. However, the short-term exposure to other pesticides was associated with a decrease in Lym of 0.32 × 10^9^/L (95% CI: −0.62, −0.02) or 5.37% (95% CI: −9.34, −1.40). Moreover, the positive association between the short-term exposure to other pesticides and neutrophil counts (Neu) was also seen. As for the parameters of the red blood cells, we found that the short-term ONs exposure was associated with the increased hematocrit (Hct), and the medium-term ONs exposure was then associated with the increased hemoglobin (Hb).

### Associations of pesticide exposures with the blood chemistry panel

As shown in Table 3, there were considerable associations between occupational pesticide exposures and nine BCP parameters. The short-term exposures to both OPs and OSs were associated with the increased level of alanine aminotransferase (ALT) and aspartate aminotransferase (AST). Moreover, each kilogram increase in the short-term OSs exposure was also associated with 0.98 U/L (95% CI: −1.84, −0.13) decrease in cholinesterase (ChE), while the medium-term OSs exposure was positively associated with an increase in total protein (TP) of 0.39 g/L (95% CI: 0.01, 0.76). In addition, the medium-term ONs exposure was also found to be positively associated with TP. In terms of parameters of renal function, only a positive association between the medium-term OSs exposure and blood urea nitrogen (BUN) was seen. Moreover, we also found that the medium-term OSs exposure was also associated with a decrease in vitamin B_12_ (VB_12_) of 26.89 ng/L (95% CI: −51.73, −2.06). The magnitudes of serum sodium (Na) and glucose (GLU) were only seen to be positively associated with the medium-term ONs exposure. PYRs was only associated with the increased C-reactive protein (CRP) following the medium-term exposure.

### Associations of pesticide exposures with peripheral nerve conduction

Table 4 shows the results for CNCS parameters associated with occupational pesticide exposures. OPs exposure was the most commonly associated with the CNCS parameters. The medium-term OPs exposure was associated with the reductions in the motor (UNMCV) and sensory conduction velocity (UNSCV) of the ulnar nerve of 0.20 m/s (95% CI: −0.39, −0.02) and 0.26 m/s (95% CI: −0.45, −0.06), respectively. Moreover, we also found that the medium-term OPs exposure was also negatively associated with the motor conduction velocity (TNMCV) and the compound muscle action potential amplitudes of the tibial nerve at the proximal (TNPCMAPA) and distal ends (TNDCMAPA). In addition, the decreased motor conduction velocity of the common peroneal nerve (PNMCV), and the decreased compound muscle action potential amplitudes of the median nerve at the distal ends (MNDCMAPA) were also seen following the medium- and short-term OPs exposure, respectively. However, OSs was only associated with a decrease in the sensory nerve action potential amplitude of the median nerve (MNSNAPA) of 2.11 mV (95% CI: −3.75, −0.46) and ulnar nerve (UNSNAPA) of 1.26 mV (95% CI: −2.47, −0.04) follow the short-term exposure. In contrast, the associations of ONs with the CNCS parameters were only seen following the medium-term exposure. For example, a decrease in the sensory conduction velocity (MNSCV) and the action nerve potential amplitude (MNSNAPA) of the median nerve were associated with the medium-term ONs exposure. Moreover, the medium-term ONs exposure was also associated with a decrease in the motor conduction velocity of the tibial nerve of 0.24 m/s (95% CI: −0.47, −0.01), and a decrease in the sensory conduction velocity of the sural nerve of 0.33 m/s (95% CI: −0.64, −0.01). PYRs was only associated with the decreased UNSNAPA following the short-term exposure, while other pesticides were only associated with the decreased UNSCV following the short-term exposure, and the decreased sensory nerve action potential amplitude of the sural nerve (SNSNAPA) following the medium-term exposure.

## Discussion

Our results demonstrated that occupational exposures to pesticides with heterogeneous chemical structures were associated with farmer health in different patterns, and the association between a specific group of pesticides and farmer health also differed between the short- and medium-term exposures. Although we found associations between multiple groups of pesticides and several CBC parameters, it was not enough to provide strong evidence of hematological disorders. However, occupational exposures to OPs, OSs and ONs might be associated with liver damage, renal dysfunction, the loss of vitamins and the increased glucose. In addition, the associations of OPs and ONs with peripheral nerve conduction among farmers were much more common and severe than that of OSs and PYRs.

In this study, OPs accounted for the largest proportion (approximately 48.5%) of the total amount of applied pesticides, which was similar to the findings of previous studies^23^,^24^. Most OPs applied by farmers were moderately, highly, and even extremely toxic (e.g., dichlorvos, chlorpyrifos, omethoate, and terbufos). Here it appeared that the adverse health effects of OPs exposure were the most severe. Our results demonstrated that the short-term OPs exposure might induce acute liver damage, which was consistent with the previous human and animal studies^25^,^26^. Moreover, our results also provided evidence that occupational OPs exposure was the most commonly associated with chronic neurotoxicity. The reduced nerve conduction velocities and the compound muscle action potential amplitudes associated with the medium-term OPs exposure suggested a increased risk of demyelination and axonal damage, respectively. In this study, the adverse effects of the medium-term OPs exposure on the ulnar and tibial nerves seemed to be more severe than on the other nerves. Similar findings were also reported in substantial previous epidemiological studies^27^,^28^,^29^.

As for OSs exposure, the decreased PDW in the short term was not a strong evidence of hematological disorders, since there was no association between the short-term OSs exposure and mean volume of platelets (MPV). Similar situation was also found on the association between the medium-term OSs exposure and Lym. In terms of BCP parameters, OSs might impair farmers’ hepatic function due to its associations with ALT, AST and ChE following the short-term exposure, and with TP following the medium-term exposure. Consistently, the liver damages associated with several most commonly used organosulfur pesticides were previously reported in the animal studies^30^,^31^,^32^. Here our results also showed that the medium-term OSs exposure might also increase the risk of chronic renal impairment, which was also consistent with the conclusions previously^33^,^34^. Moreover, OSs might lead to the loss of VB_12_ following the medium-term exposure. In terms of the neurotoxic effects, our results suggested that the short-term OSs exposure might induce acute axonal damage of the median and ulnar nerves. However, the neurotoxicity of OSs exposure was inappreciable compared with that of OPs exposure.

In this study, farmers’ exposure to ONs might increase Hct only in the short term and increase Hb only in the medium term. However, none associations between ONs exposure and these two hematological parameters were simultaneously seen in the same duration, which failed to provide convincing evidence of hematological disorders. Similar to OSs exposure, the medium-term ONs exposure might also induce chronic renal impairment indicated by the increased TP. Moreover, the medium-term ONs exposure might increase the level of GLU, which was consistent with the findings in previous rat or fish studies^35^,^36^. In addition, our results showed that the neurotoxicity of ONs exposure was second only to that of OPs exposure. In particular, the reduced sensory conduction velocity and sensory nerve action potential amplitude of the median nerve indicated the demyelination and axonal damage. Note that these findings were in accordance with the previously documented neurotoxicity of carbamate and neonicotinoid insecticides^37^.

Occupational PYRs exposure appeared to be the least toxic to humans. The increased CRP associated with the medium-term PYRs exposure might be a useful biomarker for inflammation. However, similar result was not found in the previous studies. Although the short-term PYRs exposure was also associated with the reduced UNSNAPA, its neurotoxicity was much slighter than that of OPs and ONs exposures. Similarly, a previous study indicated that the PYRs exposure during pregnancy might not induce considerable neurotoxic effect on the infants^38^. However, the estimated least neurotoxicity of PYRs exposure in this study might also be attributed to the low dose of application among farmers since the effect was probably dose-dependent.

There are several strengths and limitations to be considered. We included different groups of pesticides with heterogeneous chemical structures in an integrated framework, which allowed us to analyze the net health effect of each group of pesticides simultaneously. Moreover, subject to the application dose and duration, farmers’ exposure to pesticides may not induce the observable clinical symptoms, but the sub-clinical damages often ignored in the previous studies may probably occur following pesticide exposure. Thus, the analyses on the dose-dependent effects of pesticide exposure on farmer health, rather than the associations between pesticide exposure and the RR, OR and RD, could produce more useful findings. However, compared with some previous epidemiological studies, this study had a relatively small sample size, which might not perfectly avoid the biased estimation of the associations between pesticide exposure and health parameters. Moreover, our analyses did not include farmers’ exposure to other non-pesticide toxic substances or pesticides applied by their neighbors as the confounders in the estimation models.

In conclusion, occupational exposures to pesticides with heterogeneous chemical structures may induce different adverse health effects. The short- and medium-term OPs exposure may mainly associated with liver damage and peripheral nerve impairment, respectively, while OSs exposure may induce both liver damage and renal dysfunction. The neurotoxicity of ONs exposure is second only to OPs exposure in addition to its potential liver damage and the induced alterations in glucose. In comparison, the estimated results show that PYRs may be the least toxic in terms of the low dose of application.

## Additional Information

**How to cite this article**: Huang, X. *et al.* Association between occupational exposures to pesticides with heterogeneous chemical structures and farmer health in China. *Sci. Rep.* **6**, 25190; doi: 10.1038/srep25190 (2016).

## Supplementary Material

Supplementary Information

## Figures and Tables

**Table 1:** Summary of demographic characteristics and exposure levels (*n* = 224).

Variable	Mean ± SD or *n* (%)
Age (year)	51.77 ± 10.05
Body mass index (kg/m^2^)	23.53 ± 3.64
Female (yes = 1, no = 0)	60 (26.8)
Smoking habit (yes = 1, no = 0)	105 (46.9)
Alcohol consumption (yes = 1, no = 0)	95 (42.4)
Total pesticide exposure (kg)	4.54 ± 5.52
Short-term exposures (kg)	
OPs	0.09 ± 0.27
OSs	0.08 ± 0.20
ONs	0.04 ± 0.14
PYRs	0.01 ± 0.03
Others	0.06 ± 0.32
Medium-term exposures (kg)	
OPs	2.11 ± 3.36
OSs	0.51 ± 1.21
ONs	1.15 ± 2.13
PYRs	0.07 ± 0.19
Others	0.41 ± 0.85

SD: standard deviation.

**Table 2:** Association between pesticide exposure and complete blood cell counts (*n* = 214).

Group	Parameter (Unit)	Short term		Medium term	
		β_1_ (95% CI)	*p*-value	β_2_ (95% CI)	*p*-value
OSs	PDW (fL)	−0.38 (−0.68, −0.08)^*^	0.01	0.01 (−0.04, 0.06)	0.76
	Lym (10^9^/L)	0.03 (−0.41, 0.46)	0.90	0.07 (0.00, 0.14)^*^	<0.05
ONs	Lym (10^9^/L)	0.58 (0.00, 1.15)^*^	<0.05	−0.01 (−0.05, 0.03)	0.55
	Hb (g/L)	7.90 (−1.96, 17.75)	0.12	0.66 (0.02, 1.30)^*^	0.04
	Hct (%)	8.11 (4.51, 11.70)^**^	0.00	0.20 (−0.02, 0.41)	0.06
PYRs	PDW (fL)	−2.91 (−5.43, −0.39)^*^	0.02	0.32 (0.02, 0.63)^*^	0.04
Others	Neu (10^9^/L)^b^	0.83 (0.19, 1.47)^*^	0.01	−0.08 (−0.35, 0.20)	0.59
	Neup (%)	5.71 (1.50, 9.92)^**^	<0.01	−1.16 (−2.95, 0.63)	0.20
	Lym (10^9^/L)	−0.32 (−0.62, −0.02)^*^	0.03	0.12 (−0.01, 0.25)	0.06
	Lymp (%)	−5.37 (−9.34, −1.40)^**^	<0.01	1.23 (−0.45, 2.92)	0.15

The models were adjusted for age, sex, body mass index, smoking habit, alcohol consumption, and two province dummy variables for Guangdong and Jiangxi. The number of observations for Neu and Neup is 213 due to data non-availability of indices. CI: confidence interval.

**Table 3:** Association between pesticide exposure and blood chemistry panel (*n* = 214).

Group	Parameter (Unit)	Short term		Medium term	
		β_1_ (95% CI)	*p*-value	β_2_ (95% CI)	*p*-value
OPs	ALT (U/L)	11.93 (5.14, 18.72)^**^	<0.01	0.04 (−0.46, 0.54)	0.88
	AST (U/L)	9.79 (5.07, 14.51)^**^	<0.01	−0.06 (−0.41, 0.29)	0.74
OSs	ALT (U/L)	13.15 (4.65, 21.65)^**^	<0.01	0.88 (−0.52, 2.28)	0.22
	AST (U/L)	8.84 (2.89, 14.79)^**^	<0.01	0.92 (−0.07, 1.90)	0.07
	ChE (10^3^ U/L)	−0.98 (−1.84, −0.13)^*^	0.02	0.06 (−0.08, 0.20)	0.39
	TP (g/L)	−1.80 (−4.09, 0.49)	0.12	0.39 (0.01, 0.76)^*^	<0.05
	BUN (mmol/L)	−0.48 (−1.48, 0.52)	0.35	0.22 (0.06, 0.38)^**^	<0.01
	VB_12_ (ng/L)	53.54 (−98.78, 205.80)	0.49	−26.89 (−51.73, −2.06)^*^	0.03
ONs	TP (g/L)	−0.58 (−3.61, 2.45)	0.71	0.22 (0.02, 0.41)^*^	0.03
	Na (mmol/L)	−1.51 (−4.09, 1.08)	0.25	0.19 (0.02, 0.36)^*^	0.03
	GLU (mmol/L)	−0.23 (−1.05, 0.60)	0.59	0.05 (0.00, 0.11)^*^	<0.05
PYRs	CRP (mg/L)	−27.56 (−59.86, 8.74)	0.14	4.33 (0.18, 8.49)^*^	0.04

The models were adjusted for age, sex, body mass index, smoking habit, alcohol consumption, and two province dummy variables for Guangdong and Jiangxi. CI: confidence interval.

**Table 4:** Association between pesticide exposure and nerve conduction (*n* = 218).

Group	Parameter (Unit)	Short term		Medium term	
		β_1_ (95% CI)	*p*-value	β_2_ (95% CI)	*p*-value
OPs	MNDCMAPA (mV)	−1.77 (−3.47, −0.07)^*^	0.04	0.06 (−0.07, 0.19)	0.36
	UNMCV (m/s)	0.64 (−1.80, 3.08)	0.61	−0.20 (−0.39, −0.02)^*^	0.03
	UNSCV (m/s)	1.31 (−1.18, 3.80)	0.30	−0.26 (−0.45, −0.06)^**^	<0.01
	TNMCV (m/s)	−0.20 (−2.32, 1.92)	0.85	−0.21 (−0.37, −0.04)^*^	0.01
	TNPCMAPA (mV)	−0.34 (−1.88, 1.21)	0.67	−0.13 (−0.25, −0.01)^*^	0.03
	TNDCMAPA (mV)	0.07 (−1.92, 2.06)	0.94	−0.18 (−0.33, −0.03)^*^	0.02
	PNMCV (m/s)	−0.58 (−2.49, 1.33)	0.55	−0.27 (−0.41, −0.12)^**^	0.00
OSs	MNSNAPA (mV)	−2.11 (−3.75, −0.46)^*^	0.01	0.19 (−0.08, 0.46)	0.17
	UNSNAPA (mV)	−1.26 (−2.47, −0.04)^*^	0.04	−0.06 (−0.26, 0.14)	0.56
ONs	MNSCV (m/s)	−1.46 (−5.88, 2.97)	0.52	−0.44 (−0.73, −0.14)^**^	0.00
	MNSNAPA (mV)	1.13 (−1.02, 3.27)	0.30	−0.21 (−0.36, −0.07)^**^	<0.01
	TNMCV (m/s)	−3.06 (−6.61, 0.49)	0.09	−0.24 (−0.47, −0.01)^*^	0.05
	SNSCV (m/s)	−3.05 (−7.83, 1.74)	0.21	−0.33 (−0.64, −0.01)^*^	0.04
PYRs	UNSNAPA (mV)	−11.54 (−20.49, −2.59)^*^	0.01	0.20 (−1.02, 1.42)	0.75
Others	UNSCV (m/s)	−2.27 (−4.40, −0.13)^*^	0.04	0.43 (−0.45, 1.30)	0.34
	SNSNAPA (mV)	0.76 (−1.93, 3.45)	0.58	1.22 (−2.32, −0.12)^*^	0.03

The models were adjusted for age, sex, body mass index, smoking habit, alcohol consumption, and two province dummy variables for Guangdong and Jiangxi. CI: confidence interval.
