# Supplementary material for: Association between occupational exposures to pesticides with heterogeneous chemical structures and farmer health in China
Source: Sci Rep. 2016 Apr 27;6:25190. doi: 10.1038/srep25190 (PMC4846826; doi:10.1038/srep25190)
Supplement: Supplementary Information [file srep25190-s1.pdf]

Supplemental Material

Association between occupational exposures to pesticides with heterogeneous chemical structures and farmer health in China

Authors:

Xusheng Huang, Chao Zhang, Ruifa Hu, Yifan Li, Yanhong Yin, Zhaohui Chen, Jinyang Cai, and Fang Cui

Table of Contents

Table S1. Definition and description of health parameters.

Table S2. Classification of five groups of pesticides.

Table S1. Definition and description of health parameters.

Health parameter	Definition	Unit	First examination		Second examination	
			Mean ±SD	Range	Mean ±SD	Range
Complete blood cell counts						
WBC	White blood cell	10^9/L	6.17 ±1.92	(3.4, 24.3)	6.30 ±1.83	(2.47, 14.44)
Neu	Neutrophil	10^9/L	3.62 ±1.04	(1.2, 6.8)	3.73 ±1.30	(1.3, 10)
Neup	Neutrophil percentage	%	59.05 ±8.05	(19.3, 79.2)	58.75 ±8.08	(40.1, 82.3)
Lym	Lymphocyte	10^9/L	2.10 ±1.31	(0.8, 18.8)	2.02 ±0.81	(0.79, 8.44)
Lymp	Lymphocyte percentage	%	33.48 ±7.76	(13.2, 77.4)	32.49 ±8.12	(7.1, 58.4)
Mon	Monocyte	10^9/L	0.45 ±0.17	(0.15, 1.3)	0.17 ±0.12	(0, 0.67)
Monp	Monocyte percentage	%	7.46 ±2.33	(3, 19)	2.52 ±1.47	(0.1, 7)
RBC	Red blood cell	10^{12}/L	4.79 ±0.55	(3.16, 7.05)	4.44 ±0.54	(2.95, 6.17)
Hb	Hemoglobin	g/L	145.31 ±15.21	(68, 179)	140.24 ±16.06	(83, 176)
Hct	Hematocrit	%	43.06 ±4.94	(0.4, 51.6)	41.72 ±4.41	(27.9, 53.6)

MCV	Mean corpuscular volume	fL	90.65±5.83	(67, 109)	94.33±7.17	(68.4, 109.6)
MCH	Mean corpuscular hemoglobin	pg	30.52±2.55	(18, 38)	31.70±2.68	(19.3, 39.3)
MCHC	Mean corpuscular hemoglobin concentration	g/L	335.81±13.26	(240, 372)	336.08±15.19	(274, 401)
RDW_CV	Red cell distribution width coefficient of variation	%	12.70±0.95	(11.2, 19)	12.44±1.16	(10.1, 20)
PLT	Platelet count	10^9/L	208.58±59.22	(16, 328)	212.66±72.63	(27, 448)
MPV	Mean volume of platelets	fL	11.0±1.12	(8.8, 13.6)	10.19±1.61	(7.7, 15.3)
PDW	Platelet distribution width	fL	14.01±1.95	(9.9, 20.7)	15.99±0.39	(15, 18)
Blood chemistry panel						
**Hepatic function**						
ALT	Alanine aminotransferase	U/L	24.54±13.45	(4.00, 106.00)	20.18±11.79	(4.00, 122.00)
AST	Aspartate aminotransferase	U/L	26.36±8.91	(13.00, 72.00)	23.01±12.08	(12.00, 172.0)
ChE	Cholinesterase	10^3U/L	8.09±1.66	(3.24, 12.31)	8.71±1.73	(4.65, 13.53)

TP	Total protein	g/L	69.36 ±3.90	(62.00, 79.00)	76.81 ±4.84	(63.70, 92.80)
**Renal function**						
BUN	Blood urea nitrogen	mmol/L	5.52 ±1.42	(3.00, 11.26)	5.15 ±1.31	(1.90, 11.40)
Cr	Creatinine	μmol/L	75.52 ±18.07	(44.0, 122.0)	72.99 ±14.05	(44.0, 124.0)
**Electrolytes**						
Na	Serum sodium	mmol/L	140.71 ±2.37	(136.2, 144.9)	141.97 ±2.49	(134.0, 151.0)
K	Serum potassium	mmol/L	4.45 ±0.46	(3.60, 5.20)	4.68 ±0.67	(3.68, 9.20)
P	Inorganic phosphorus	mmol/L	1.31 ±0.28	(0.88, 2.20)	1.18 ±0.15	(0.77, 1.62)
**Vitamins**						
VB_{12}	Vitamin B12	ng/L	480.94 ±218.3	(109.7, 945.7)	494.9 ±248.44	(142, 1550)
VB_9	Folic acid	μg/L	10.52 ±4.04	(3.25, 19.17)	8.29 ±4.42	(2.97, 22.00)
**Other**						
GLU	Glucose	mmol/L	4.86 ±1.00	(3.90, 14.08)	5.46 ±1.02	(4.16, 12.48)
CRP	C-reactive protein	mg/L	1.29 ±5.01	(0.00, 52.00)	1.62 ±3.63	(0.01, 37.20)

Nerve conduction						
**Velocity**						
MNMCV	Median nerve motor conduction velocity	m/s	59.76±4.46	(43.50, 73.30)	58.70±4.25	(41.70, 68.60)
UNMCV	Ulnar nerve motor conduction velocity	m/s	58.00±4.49	(46.40, 73.70)	57.18±4.11	(46.40, 68.40)
TNMCV	Tibial nerve motor conduction velocity	m/s	47.68±3.94	(35.90, 60.00)	47.68±3.85	(38.80, 63.70)
PNMCV	Common peroneal nerve motor conduction velocity	m/s	48.59±4.47	(27.00, 60.00)	48.49±4.32	(25.80, 57.10)
MNSCV	Median nerve sensory conduction velocity	m/s	58.04±6.11	(33.30, 69.60)	58.51±6.33	(32.60, 72.70)
UNSCV	Ulnar nerve sensory conduction velocity	m/s	54.33±4.53	(37.00, 65.70)	54.17±5.40	(20.00, 68.00)
SNSCV	Sural nerve sensory conduction velocity	m/s	59.58±4.98	(50.00, 75.00)	60.44±5.64	(50.00, 71.40)
**Distal motor latency**						
MNDML	Median nerve distal motor latency	ms	3.34±0.52	(2.10, 7.20)	3.45±0.67	(2.60, 8.70)
UNDML	Ulnar nerve distal motor latency	ms	2.63±0.28	(2.00, 3.60)	2.86±0.65	(2.00, 8.90)
TNDML	Tibial nerve distal motor latency	ms	3.67±0.46	(2.70, 4.80)	3.69±0.47	(2.70, 5.00)

PNDML	Common peroneal nerve distal motor latency	ms	3.73±0.63	(2.50, 6.70)	3.72±0.81	(2.70, 11.80)
**Amplitude**						
MNPCMAPA	Median nerve proximal compound muscle action potential amplitude	mV	12.20±3.23	(5.60, 20.90)	13.68±3.99	(5.70, 27.40)
MNDCMAPA	Median nerve distal compound muscle action potential amplitude	mV	12.97±3.40	(5.60, 23.00)	14.13±4.11	(6.00, 28.80)
UNPCMAPA	Ulnar nerve proximal compound muscle action potential amplitude	mV	11.33±2.29	(5.20, 18.90)	12.44±2.79	(1.00, 21.10)
UNDCMAPA	Ulnar nerve distal compound muscle action potential amplitude	mV	12.17±2.27	(6.10, 20.00)	13.07±2.71	(5.40, 23.00)
TNPCMAPA	Tibial nerve proximal compound muscle action potential amplitude	mV	11.21±3.86	(3.50, 25.00)	11.21±3.84	(3.10, 21.40)
TNDCMAPA	Tibial nerve distal compound muscle action	mV	14.31±4.74	(4.20, 26.70)	13.54±4.49	(4.30, 27.70)

	potential amplitude					
PNPCMAPA	Common peroneal nerve proximal compound muscle action potential amplitude	mV	6.50±3.12	(0.10, 20.20)	6.79±3.27	(0.10, 19.80)
PNDCMAPA	Common peroneal nerve distal compound muscle action potential amplitude	mV	7.17±3.32	(0.10, 19.10)	7.37±3.34	(0.10, 18.50)
MNSNAPA	Median sensory nerve action potential amplitude	mV	7.67±3.17	(1.20, 21.00)	8.41±3.35	(2.00, 21.00)
UNSNAPA	Ulnar sensory nerve action potential amplitude	mV	5.84±2.49	(1.00, 17.00)	6.29±2.65	(0.70, 18.00)
SNSNAPA	Sural sensory nerve action potential amplitude	mV	15.24±6.91	(4.20, 44.00)	16.22±7.60	(4.70, 69.00)

Table S2. Classification of five groups of pesticides.

Group name	Chemical name	
	Chinese	English
**Organophosphates**	乙酰甲胺磷	Acephate
	毒死蜱	Chlorpyrifos
	二嗪磷	Diazinon
	敌敌畏	Dichlorvos
	乐果	Dimethoate
	稻瘟净	EBP
	三乙磷酸铝	Fosetyl-aluminium
	草甘膦	Glyphosate
	水胺硫磷	Isocarbophos
	马拉硫磷	Malathion
	氧乐果	Omethoate
	对硫磷	Parathion
	稻丰散	Phenthoate
	甲拌磷	Phorate
	亚胺硫磷	Phosmet
	辛硫磷	Phoxim
	丙溴磷	Profenofos
	特丁硫磷	Terbufos
	甲基立枯磷	Tolclofos-methyl

	三唑磷	Triazophos
	敌百虫	Trichlorfon
**Organosulfurs**	苄嘧磺隆	Bensulfuron-methyl
	杀虫双	Bisultap
	烯草酮	Clethodim
	二甲基二硫醚	Dithioether
	乙蒜素	Ethylicin
	敌磺钠	Fenaminosulf
	稻瘟灵	Isoprothiolane
	代森锰锌	Mancozeb
	硝磺草酮	Mesotrione
	代森联	Metiram
	甲磺隆	Metsulfuron-methyl
	杀虫单	Monosultap
	烟嘧磺隆	Nicosulfuron
	五氟磺草胺	Penoxsulam
	炔螨特	Propargite
	丙森锌	Propineb
	噻菌茂	Saijunmao
	噻唑锌	Saizuoxin
	噻吩磺隆	Thifensulfuron-methyl
	甲基硫菌灵	Thiophanate-methyl

	福美双	Thiram
	苯磺隆	Tribenuron-methyl
	代森锌	Zineb
	福美锌	Ziram
**Organonitrogens**	啶虫脒	Acetamiprid
	乙草胺	Acetochlor
	甲草胺	Alachlor
	莠去津	Atrazine
	噻霉酮	Benziothiazolinone
	叶枯唑	Bismerthiazol
	溴菌腈	Bromothalonil
	噻嗪酮	Buprofezin
	丁草胺	Butachlor
	仲丁灵	Butralin
	多菌灵	Carbendazim
	克百威	Carbofuron
	丁硫克百威	Carbosulfan
	氯虫苯甲酰胺	Chlorantraniliprole
	灭幼脲	Chlorbenzuron
	氟啶脲	Chlorfuazuron
	氯溴异氰尿酸	Chloroisobromine cyanuric acid
	溴氰虫酰胺	Cyantraniliprole

氰氟草酯	Cyhalofop-butyl
霜脲氰	Cymoxanil
灭蝇胺	Cyromazine
苯醚甲环唑	Difenoconazole
除虫脲	Diflubenzuron
烯酰吗啉	Dimethomorph
烯唑醇	Diniconazole
乙嘧酚	Ethirimol
噁唑菌酮	Famoxadone
仲丁威	Fenobucarb
唑螨酯	Fenpyronximate
氟虫腈	Fipronil
咯菌腈	Fludioxonil
氟吗啉	Flumorph
乙羧氟草醚	Fluoroglycofen-ethyl
氟硅唑	Flusilazole
氟虫双酰胺	Fuchongshuangxianan
氟酰胺	Fuchongshuangxianan
高效氟吡甲禾灵	Haloxyfop-P-methyl
噁霉灵	Hemexazol
己唑醇	Hexaconazole
氟铃脲	Hexaflumuron

噁霉灵	Hymexazol
吡虫啉	Imidacloprid
茚虫威	Indoxacarb
异菌脲	Iprodione
缬霉威	Iprovalicarb
异丙威	Isoprocarb
氰氟虫腙	Metaflumizone
甲霜灵	Metalaxyl
精甲霜灵	Metalaxyl-M
灭多威	Methomyl
异丙甲草胺	Metolachlor
速灭威	Metolcarb
盐酸吗啉胍	Moroxydine hydrochloride
腈菌唑	Myclobutanil
烟碱	Nicotine
烯啶虫胺	Nitenpyram
百草枯	Paraquat
二甲戊灵	Pendimethalin
丙草胺	Pretilachlor
咪鲜胺	Prochloraz
腐霉利	Procymidone
扑草净	Prometryn

	霜霉威	Propamocarb
	丙环唑	Propiconazol
	异丙草胺	Propisochlor
	吡蚜酮	Pymetrozine
	吡唑醚菌酯	Pyraclostrobin
	哒螨灵	Pyridaben
	嘧霉胺	Pyrimethanil
	精喹禾灵	Quizalofop-ethyl
	戊唑醇	Tebuconazole
	虫酰肼	Tebufenozide
	噻虫嗪	Thiamethoxam
	三唑酮	Triadimefon
	肟菌酯	Trifloxystrobin
	氟乐灵	Trifluralin
	辛菌胺	Xinjunan
**Pyrethroids**	顺式氯氰菊酯	Alpha-cypermethrin
	高效氯氰菊酯	Beta-cypermethrin
	联苯菊酯	Bifenthrin
	氯氟氰菊酯	Cyhalothrin
	氯氰菊酯	Cypermethrin
	溴氰菊酯	Deltamethrin
	醚菊酯	Etofenprox

	甲氰菊酯	Fenpropathrin
	氰戊菊酯	Fenvalerate
	高效氯氟氰菊酯	Lambda-cyhalothrin
**Others**	2,4-滴丁酯	2,4-D butylate
	阿维菌素	Abamectin
	印楝素	Azadirachtin
	苏云金杆菌	*Bacillus thuringiensis*
	白僵菌	Beauveria
	毒杀芬	Camphechlor
	百菌清	Chlorothanonil
	四螨嗪	Clofentezine
	乙酸铜	Copper acetate
	氢氧化铜	Copper hydroxide
	硫酸铜	Copper sulfate
	甲维盐	Emamectin benzoate
	硫丹	Endosulfan
	丁子香酚	Eugenol
	氟邦尼	Fubangni
	香菇多糖	Fungous proteoglycan
	棉铃虫核型多角体病毒	Heliothis armigera NPV
	井冈霉素	Jingangmycin
	春雷霉素	Kasugamycin

苦参碱	Matrine
嘧啶核苷类抗菌素	Midingheganleikangjunsu
宁南霉素	Ningnanmycin
喹啉铜	Oxine-copper
矿物油	Petroleum oil
申嗪霉素	Phenazino-1-carboxylic acid
多抗霉素	Polyoxin
荧光假单胞杆菌	Pseudomonas fluorescens
五氯硝基苯	Quintozene
鱼藤酮	Rotenone
松脂酸铜	Songzhisuantong
多杀霉素	Spinosad
斜纹夜蛾核型多角体病毒	Spodopteraliture NPV
链霉素	Streptomycin
硫磺	Sulfur
噻菌铜	Thiediazole copper
中生菌素	Zhongshengmycin
